# Supine Ober's Test: A Physical Examination Maneuver for Evaluating External Snapping Hip

**DOI:** 10.1002/atn2.70260

**Published:** 2026-10-23

**Authors:** Zhi‐Dong Zhao, Ling‐Xing Jiang, Yao‐Ting Wang, Ming‐Xin Wang, Chun‐Bao Li

**Affiliations:** ^1^ Senior Department of Orthopedics The Fourth Medical Center of PLA General Hospital Beijing People's Republic of China

## Abstract

Provocative detection of snapping through physical examination is critical for diagnosing external snapping hip (ESH) and intraoperatively assessing soft tissue release adequacy. The lateral decubitus Ober's test (OT) is commonly used but requires patient repositioning, bringing certain clinical inconveniences. This technical note describes a modified supine OT: patients lie supine with lower extremities extended neutrally. The examiner grasps the affected side's ankle and midcalf, maximally internally rotates/adducts the limb, passively flexes the hip/knee to hyperflexion, and then extends gradually. A positive sign is visualized/palpated snapping/gliding around the greater trochanter during flexion‐extension. Compared with classic OT, supine OT offers superior convenience and applicability, which allows for simultaneous bilateral and dynamic assessment without repositioning—particularly during surgical evaluations—and provides a crucial theoretical foundation for managing ESH in the supine position. Additionally, it shortens procedural time and improves comfort for both clinicians and patients. In conclusion, supine OT is a valuable dynamic adjunct for ESH assessment, especially for bilateral conditions, complementing traditional modalities in ESH management.

## Graphical abstract

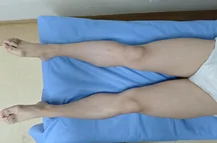

**VIDEO 1:** This video illustrates the application of the supine Ober's test (SOT) in the evaluation of external snapping hip (ESH). It covers the routine clinical examination in the outpatient setting, as well as its utility for intraoperative evaluation of bilateral ESH performed in the supine position. The patient lies supine on the examination table with both lower limbs extended in a neutral position. The affected limb was stabilized, and then the lower limb was maximally internally rotated and maintained 10° to 15° of adduction. Passive hip and knee flexion and extension were performed 2 to 3 times. Audible or palpable snapping around the greater trochanter during the hip flexion‐extension cycle was defined as positive. Otherwise, it was negative. Then, the contralateral lower limb was examined with the same method. During bilateral ESH surgery, SOT was performed to assess the snapping prior to release procedure, with a positive finding. After the release, the SOT was repeated to evaluate the adequacy of soft tissue release, and a negative SOT finding was confirmed intraoperatively. Then, the SOT was subsequently used to assess the contralateral ESH without repositioning the patient, thereby conferring greater procedural convenience. Video content can be viewed at https://doi.org/10.1002/atn2.70260.

External snapping hip (ESH), one cause of hip pain, gait abnormalities, and functional impairment, was pathologically characterized with abnormal gliding of the iliotibial band (ITB) or gluteus maximus tendon over the greater trochanter.^1^, ^2^, ^3^, ^4^, ^5^, ^6^ Theoretically, any factors that induce ITB thickening or increased tension, as well as structural abnormalities of the greater trochanter, may contribute to external hip snapping.^7^, ^8^ Clinical diagnosis of ESH primarily relies on provocative detection of snapping through physical examination.^9^ Since its first description, the lateral decubitus Ober's test (OT) has been widely used for evaluating ITB tightness and diagnosing related disorders,^10^ and it is also widely used to assess the adequacy of intraoperative soft tissue release.

However, the lateral decubitus OT requires patient repositioning during the examination process, bringing certain clinical inconveniences, especially in scenarios of dynamic evaluations during ESH releasing surgery and bedside evaluations of patients with limited mobility. Furthermore, if reliable assessments can be performed with the patient in the supine position during the operative procedure, it will achieve seamless integration of diagnostic and therapeutic workflows, thereby improving surgical efficiency.^11^

Based on clinical practice, we observed that performing the modified OT with the patient in a natural supine position can effectively induce hip contracture and snapping sensations. This modified approach simplifies the examination process and enhances its convenience and accessibility in clinical settings. Therefore, this article aims to detail the standardized operational protocol and positive diagnostic criteria for the supine OT (SOT).

## PHYSICAL EXAMINATION TECHNIQUE

Video 1 shows how to do the SOT in the outpatient setting, as well as its utility for intraoperative evaluation of bilateral ESH syndrome performed in the supine position.

Stepwise Standardization of the Supine Ober's Sign

1. The patient lies supine on the examination table with both lower limbs extended in a neutral position (Figure 1).
2. The examiner stabilizes the affected limb by grasping the area between the medial and lateral malleoli with one hand and the midcalf segment with the other hand, then maximally internally rotates the lower limb, and maintains 10° to 15° of hip adduction (Figure 2).
3. The examiner then passively performs hip and knee flexion until the hip joint reaches the maximum flexion range (Figures 3 and 4).
4. Subsequently, the hip and knee joints are passively and slowly extended to the neutral position (Figures 5 and 6).
5. The above sequence is repeated 2 to 3 times to ensure test reproducibility. A positive Ober's sign is confirmed if audible or palpable snapping/gliding around the greater trochanter is detected during the hip flexion‐extension cycle. A negative sign is indicated if no snapping sensation is observed or palpated during the maneuver. This examination can be performed in outpatient clinics, inpatient wards, and operating rooms.

**FIGURE 1:**
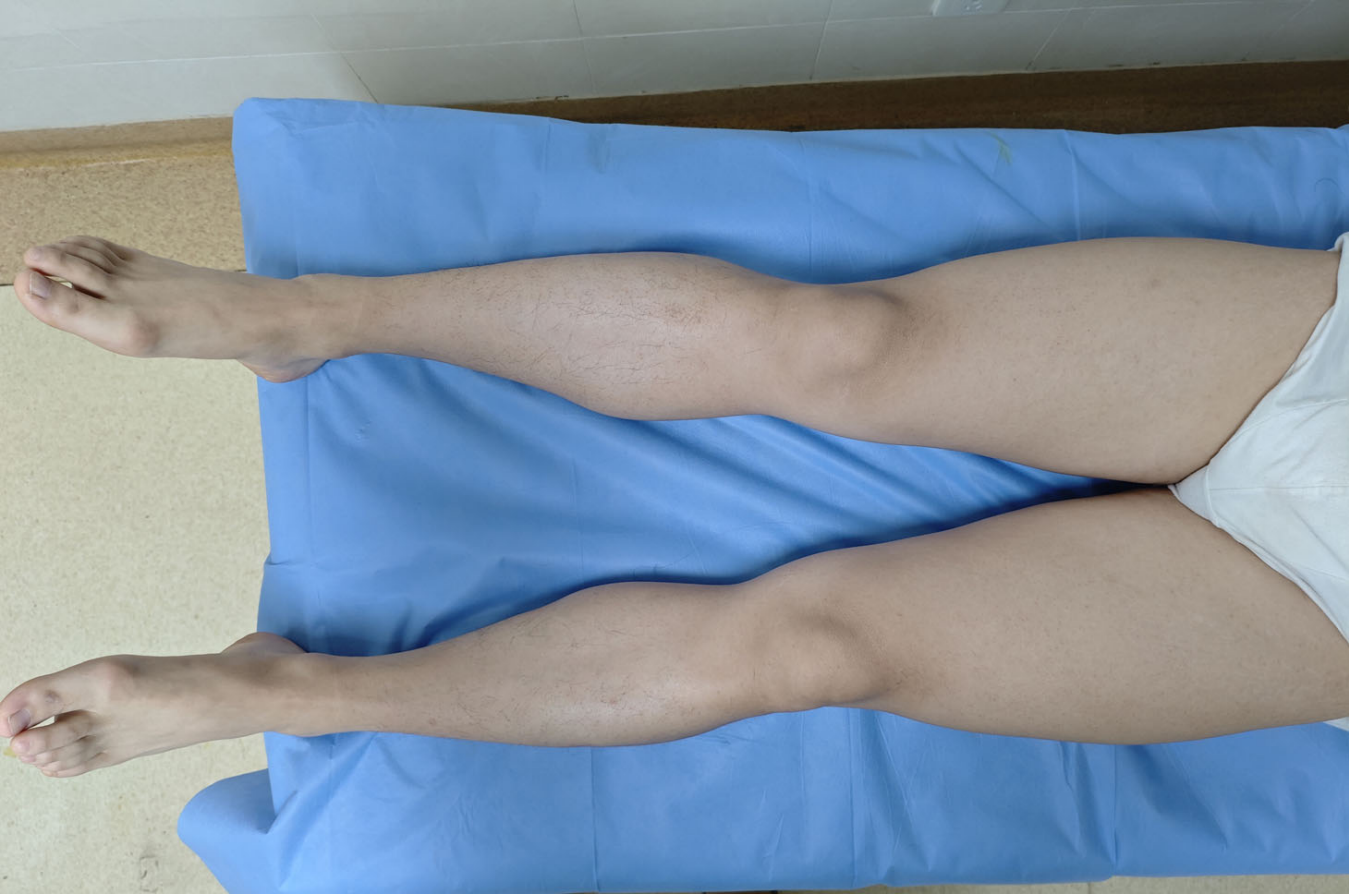
The patient lies supine on the examination table with both lower limbs extended in a neutral position.

**FIGURE 2:**
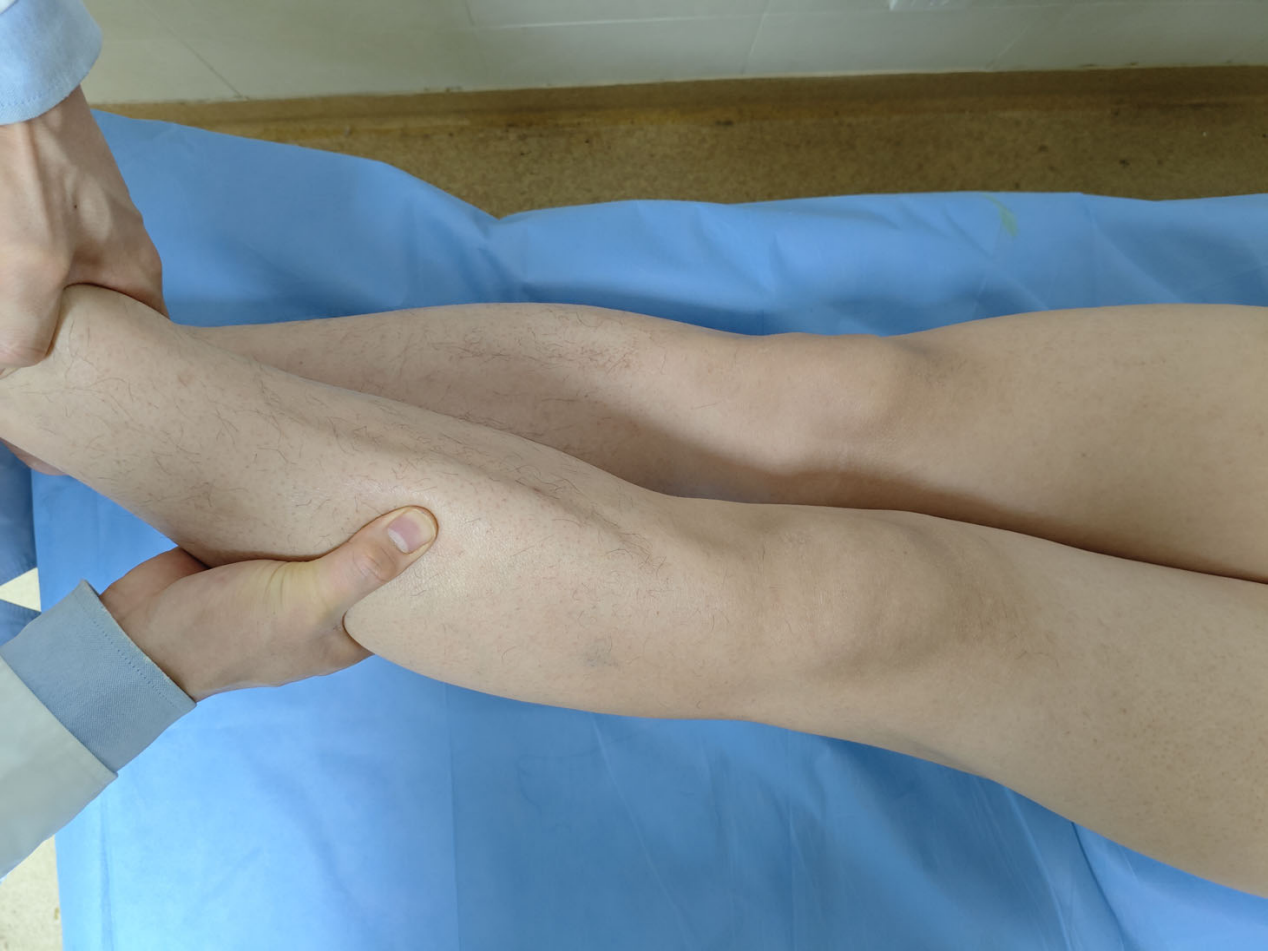
The examiner stabilizes the affected limb by grasping the area between the medial and lateral malleoli with one hand and the midcalf segment with the other hand, then maximally internally rotates the lower limb, and maintains 10°‐15° of hip adduction.

**FIGURE 3:**
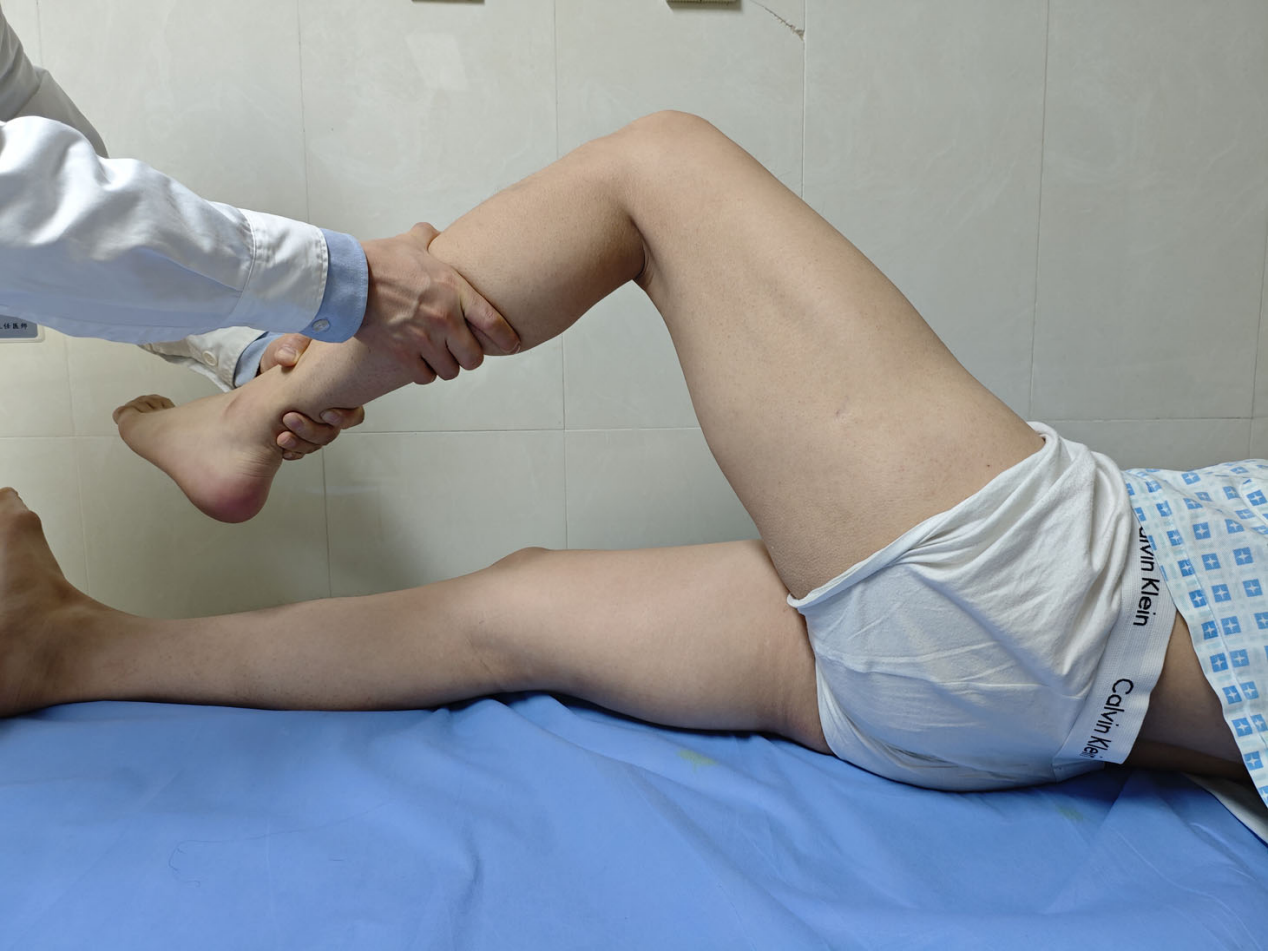
The examiner then passively performs hip and knee flexion until the hip joint reaches the maximum flexion range.

**FIGURE 4:**
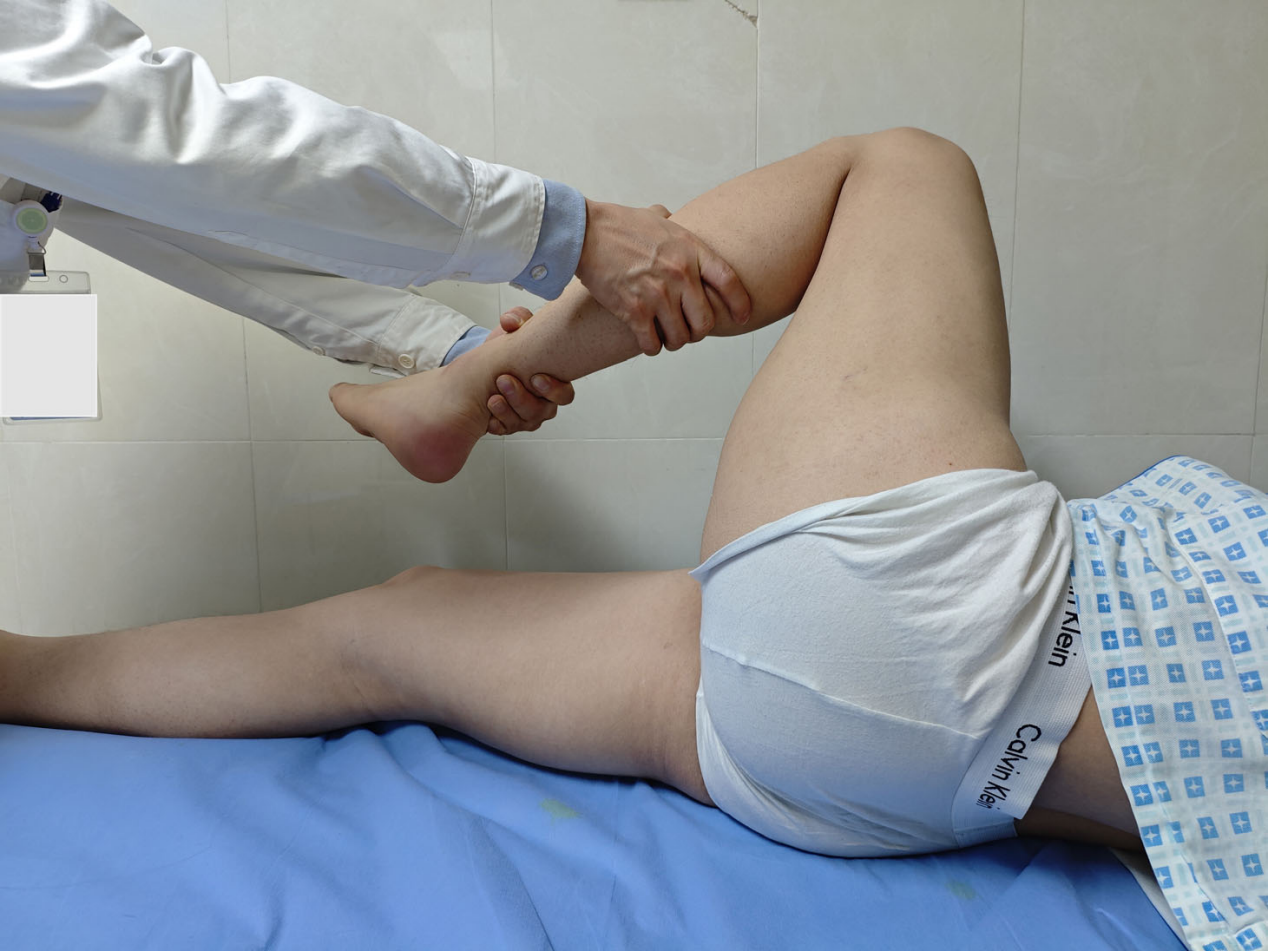
The examiner then passively performs hip and knee flexion until the hip joint reaches the maximum flexion range.

**FIGURE 5:**
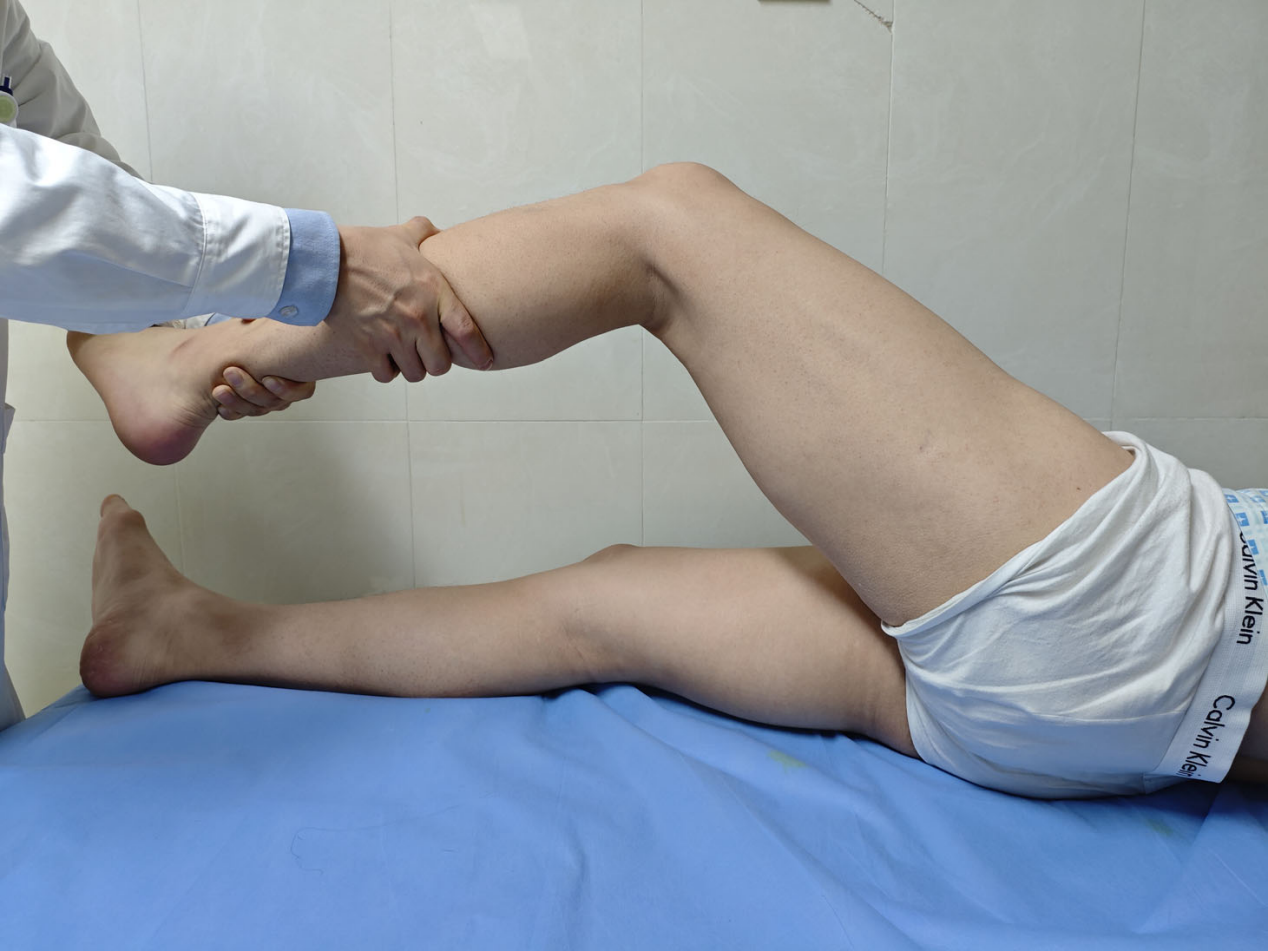
The hip and knee joints are passively and slowly extended.

**FIGURE 6:**
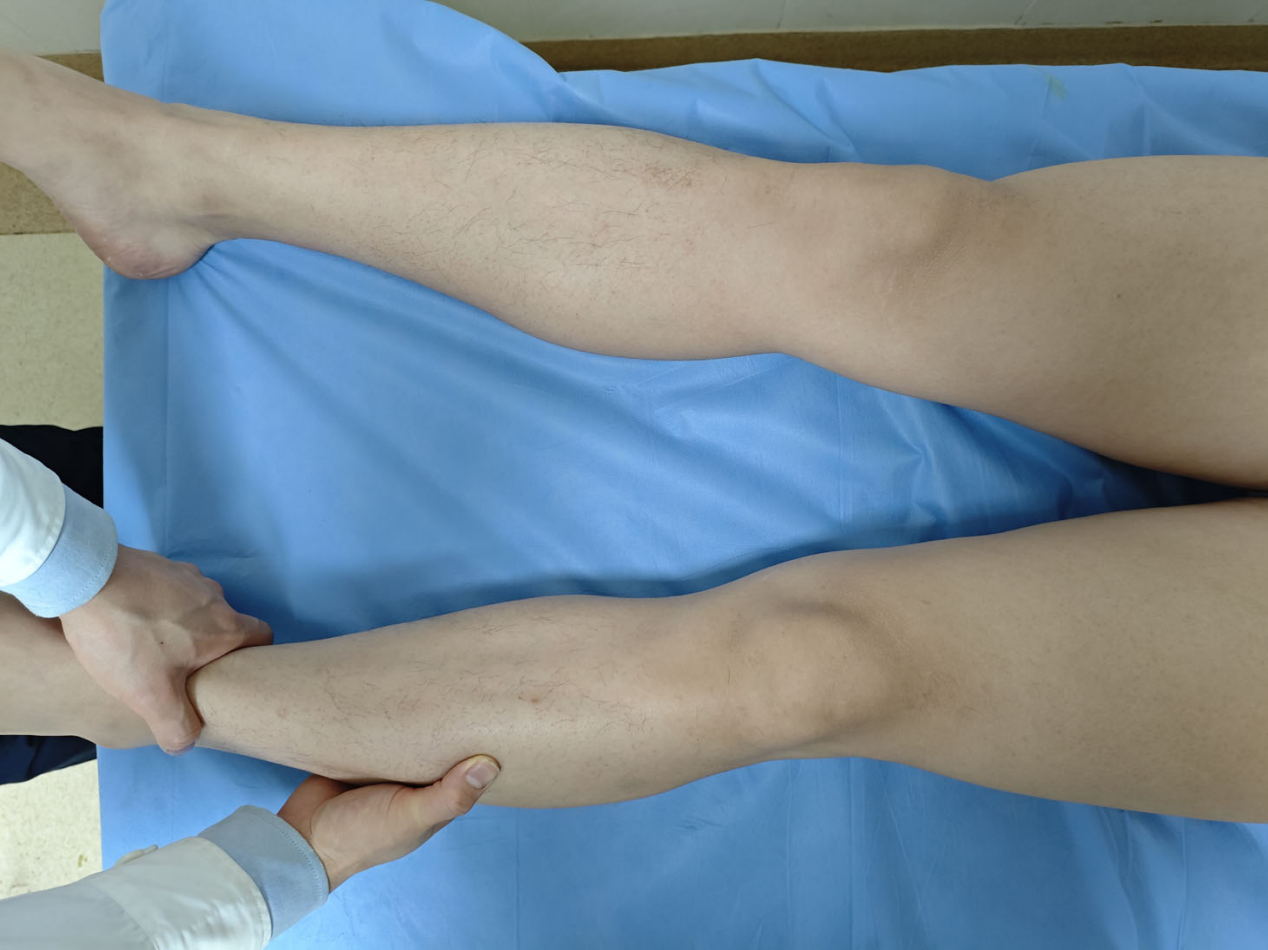
The hip and knee joints are passively and slowly extended to the neutral position.

## DISCUSSION

This technical note delineates the clinical utility of the SOT in the evaluation of ESH. Its core advantage lies in eliminating the need for patient repositioning, making it suitable for patients with severe pain or obesity in a routine outpatient setting. Moreover, the SOT is more convenient in performing simultaneous bilateral ESH releasing surgery in the supine position. By minimizing body position changes, this maneuver substantially improves patient comfort and examination tolerability, conferring greater procedural convenience.

Since its introduction in 1935, the OT has been widely employed to assess ITB tightness.^10^ Its classic description specifies that “in the absence of contracture, the thigh should be capable of adduction below the horizontal plane.” However, as a standalone physical examination maneuver, the inter‐rater reliability and reproducibility of OT have not been fully validated to date.^12^ In clinical practice, OT is extensively used as an adjunctive diagnostic tool for both gluteal muscle contracture and ESH.^13^ Although gluteal muscle contracture is pathologically defined as a myofascial disorder characterized by fibrosis of the gluteal muscles and adjacent fascia, the increased soft tissue tension associated with gluteal muscle contracture can be transmitted to the ITB via tendinous connections, thereby inducing snapping over the greater trochanter region.^14^ It is noteworthy that Willett et al. revealed that a positive Ober's sign indicates potential contracture of the ITB or tensor fasciae latae, but it has limited specificity for the diagnosis of ESH,^15^ warranting its use as a supportive rather than a definitive diagnostic sign. In 2014, de Amorim Cabrita et al.^16^ reported a supine physical examination maneuver for diagnosing ESH, yet its diagnostic efficacy and reproducibility remain to be further verified.

The SOT achieves ITB tension modulation and snapping provocation without patient repositioning through a combined sequence of hip adduction, internal rotation, and subsequent flexion in the supine position. This design not only preserves the pathophysiological rationale underlying the traditional OT but also addresses the unmet clinical need for real‐time intraoperative assessment. Of note, the diagnostic performance of SOT still requires validation with high‐level clinical evidence. Additionally, examination results may be influenced by the clinician's level of experience. Future research should design rigorous diagnostic accuracy studies to directly compare the SOT with the conventional lateral decubitus OT, using imaging findings or arthroscopic observations as the reference standard, to obtain higher‐level evidence supporting its clinical application. The pearls and pitfalls of SOT are shown in Table 1. The advantages and disadvantages of SOT are shown in Table 2.

**TABLE 1:** Pearls and Pitfalls of SOT

**Steps**	**Pearls**	**Pitfalls**
1	It is crucial to ensure pelvic stability and adequate support of the contralateral lower limb during the examination. Pelvic tilting can alter the baseline tension of the ITB through the fascial chain, thereby causing false‐negative test results	False‐negative results: Insufficient hip internal rotation (<25°) or inadequate hip flexion (<100°) may lead to missed diagnoses, especially for mild cases with subtle snapping. Severe joint capsule contracture or concurrent FAI with restricted range of motion can also result in false‐negative findings
2	Multimodal assessment: In outpatient settings, combining SOT with other complementary tests (e.g., the cross‐legged test) can improve the diagnostic accuracy of ESH	Anesthetic effect: Perioperative muscle relaxation under general anesthesia may attenuate the snapping phenomenon, potentially leading to misjudgment of the adequacy of intraoperative soft tissue release
3	Intraoperative application: Place a soft cushion under the affected buttock to slightly elevate the hip joint, which increases the range of passive hip adduction/internal rotation and facilitates the performance of the maneuver	
4	Dynamic arthroscopic assessment: Intraoperatively, SOT can be performed dynamically under arthroscopic visualization, enabling direct correlation between the palpable/audible snapping and the arthroscopically observed gliding of the ITB over the greater trochanter	

ESH, external snapping hip; FAI, femoroacetabular impingement; ITB, iliotibial band; SOT, supine Ober's test.

**TABLE 2:** Advantages and Disadvantages of Supine OT

**Advantages**	**Disadvantages**
Eliminating the need for repeated patient repositioning	The supine position does not allow direct visualization of the ITB from a superior‐to‐inferior perspective, making it difficult to detect subtle snapping or gliding sensations. Therefore, full communication with the patient is essential during the examination to elicit subjective symptoms
Single position for bilateral examination	Like all dynamic provocative tests, it may yield false‐negative results, which should be interpreted in conjunction with the comprehensive clinical context rather than in isolation
Fulfills the requirements of both preoperative diagnosis and intraoperative assessment simultaneously	Diagnostic efficacy to be further validated: Although preliminary clinical applications have shown promising results, additional prospective studies are required to clarify its sensitivity and specificity in comparison with the conventional OT
Intraoperative assessment is not restricted by the surgical position, facilitating anesthesia management and perioperative patient care	
Obviates the need for patient repositioning, thereby shortening surgical duration and reducing the risk of surgical site infection	

ITB, iliotibial band; OT, Ober's test.

The SOT is a practical, multiscenario physical examination maneuver suitable for the evaluation of ESH. It can be applied both for diagnostic purposes and for intraoperatively assessing surgical outcomes, showing distinct advantages in cases of bilateral ESH and supine arthroscopic hip surgeries. Thus, it serves as a valuable complementary examination tool, providing clinicians with a more flexible and efficient option in specific clinical contexts.

## DISCLOSURES

The authors (Z‐D.Z., L‐X.J., Y‐T.W., M‐X.W., C‐B.L.) declare that they have no known competing financial interests or personal relationships that could have appeared to influence the work reported in this article.

## FUNDING

This work was supported by Beijing Natural Science Foundation (No. L252061).
